# A Formal Methodology to Design and Deploy Dependable Wireless Sensor Networks

**DOI:** 10.3390/s17010019

**Published:** 2016-12-23

**Authors:** Alessandro Testa, Marcello Cinque, Antonio Coronato, Juan Carlos Augusto

**Affiliations:** 1 Ministero dell’Economia e delle Finanze, Rome 00187, Italy; alessandro.testa@tesoro.it; 2 Dipartimento di Ingegneria Elettrica e delle Tecnologie dell’Informazione, University of Naples “Federico II”, Naples 80125, Italy; macinque@unina.it; 3 CNR-ICAR, Naples 80131, Italy; 4 Department of Computer Science and R.G. on Development of Intelligent Environments, Middlesex University of London, London NW4 2SH, UK; j.augusto@mdx.ac.uk

**Keywords:** Wireless Sensor Networks, formal methods, dependability, metrics, modeling

## Abstract

Wireless Sensor Networks (WSNs) are being increasingly adopted in critical applications, where verifying the correct operation of sensor nodes is a major concern. Undesired events may undermine the mission of the WSNs. Hence, their effects need to be properly assessed before deployment, to obtain a good level of expected performance; and during the operation, in order to avoid dangerous unexpected results. In this paper, we propose a methodology that aims at assessing and improving the dependability level of WSNs by means of an event-based formal verification technique. The methodology includes a process to guide designers towards the realization of a dependable WSN and a tool (“ADVISES”) to simplify its adoption. The tool is applicable to homogeneous WSNs with static routing topologies. It allows the automatic generation of formal specifications used to check correctness properties and evaluate dependability metrics at design time and at runtime for WSNs where an acceptable percentage of faults can be defined. During the runtime, we can check the behavior of the WSN accordingly to the results obtained at design time and we can detect sudden and unexpected failures, in order to trigger recovery procedures. The effectiveness of the methodology is shown in the context of two case studies, as proof-of-concept, aiming to illustrate how the tool is helpful to drive design choices and to check the correctness properties of the WSN at runtime. Although the method scales up to very large WSNs, the applicability of the methodology may be compromised by the state space explosion of the reasoning model, which must be faced by partitioning large topologies into sub-topologies.

## 1. Introduction and Motivation

Wireless Sensor Networks (WSNs) [1] are being increasingly used in critical application scenarios where the level of trust on WSNs becomes an important factor, affecting the success of industrial WSN applications. The extensive use of this kind of network stresses the need to verify their dependability—dependability is defined as the ability of a system to deliver a service that can justifiably be trusted [2]. It is an integrated concept encompassing the attributes of reliability, availability, maintainability, safety, and integrity—not only at design time to prevent wrong design choices but also at runtime in order to make a WSN more robust against failures that may occur during its operation.

Typical critical scenarios are environmental monitoring (e.g., detection of fires in forests [1]), structural monitoring of civil engineering structures [3], health monitoring (in medical scenarios) [4] and patient monitoring [5,6] by means of Ambient Intelligence (AmI [7]) systems. For example, in the case of remote patient monitoring, alerts must be raised and processed within a temporal threshold; functional incorrectness or runtime failures may result in catastrophic consequences for the patient. These types of applications are considered safety-critical and they must be designed and developed with intrinsic and stringent dependability requirements [8]. Thus, depending on the application scenarios, different dependability requirements can be defined, such as, node lifetime, network resiliency and coverage of the monitoring area.

The work presented in [9] evidenced that also in a simple deployment, a single node can be responsible for the failure of a huge piece of the network. For instance, a node that is close to the sink (i.e., the gateway of the WSN that has the role to collect all of the measures detected by the sensors) is more likely to fail due to the great demand that it is subjected to, and its failure would likely cause the isolation of a set of nodes from the sink.

Therefore, it is necessary to verify the WSNs at design time against given correctness properties, in order to increase the confidence about the robustness of the designed solution before putting it into operation. Also, in the IoT (Internet of Things) networks, the design of a dependable WSN platform has became a main focus of research. In fact, in [10], the authors propose a functional design and implementation of a complete WSN platform that can be used for a range of long-term environmental monitoring IoT applications.

Moreover, it is also important to avoid unexpected results or dangerous effects during the runtime of the WSN; this can be obtained by checking traces of events generated from the system run against the same correctness properties used at design time.

Formal methods can be used for these purposes, due to their wide adoption in the literature to verify the correctness of a system specification [11] or to perform runtime verification [12,13]. However, the practical use of formal methods for the verification of dependability properties of WSNs has received little attention, due to the distance between system engineers and formal methods experts and the need to re-adapt the formal specification to different design choices. Even if a development team were to invest on the definition of a detailed specification of WSN correctness properties, a design change (e.g., different network topology) could require one to rethink the formal specification, incurring extra undesirable costs.

To overcome these limitations, the contribution of this paper is manyfold. Specifically, we propose:

- a formal methodology to support the design and deployment of dependable WSNs both at design time (*static verification*) and at runtime (*runtime verification*);
- the definition of a unique formal specification of WSN correctness, based on the event calculus formalism, subdivided in two logical subsets: a *general correctness specification*, valid independently of the particular WSN under study, and a *structural specification* related to the properties of the target WSN (e.g., number of nodes, topology, etc.);
- the adoption of specific WSN dependability metrics, such as *connection resiliency* and *coverage*, introduced in [14], for measuring dependability degree and providing a quantitative assessment of a WSN;
- an automated verification tool, named ADVISES (AutomateD VerIfication of wSn with Event calculuS), to facilitate the adoption of the proposed approach.

The key idea of the proposed methodology is to base the verification of correctness properties on a set of specifications that can be used interchangeably at design time and at runtime. The decomposition of the specification in two sets simplifies the adoption of the approach. While general correctness specifications do not need to be adapted when changing the target WSN, structural specifications depend on the target, and are designed to be generated automatically.

The ADVISES tool facilitates the adoption of the proposed methodology by system engineers with no experience of formal methods. At design time, it can be used to perform a *robustness checking* of the target WSN, i.e., to verify the long-term robustness of the WSN (in terms of the proposed metrics) against random sequences of undesired events, useful to identify corner cases and dependability bottlenecks. At runtime, it monitors the deployed WSN. If an undesired event occurs, the tool calculates the current values of dependability metrics (e.g., raising an alarm if a given criticality level is reached) and it assesses the criticality of the network, in terms of the future hazardous scenarios that can happen, considering the new network conditions.

The proposed methodology and tool has been applied on two realistic WSNs representative of health monitoring scenarios. Whilst these scenarios are on the smaller size, they are valuable as proof-of-concept and also to explain how to use the generic problem solving approach and tool presented in this article. The case studies show how the approach is useful to deeply investigate the reasons of inefficiency and to re-target design choices. They also show how the same specification can be used at runtime to check the correct behavior of a real WSN, deployed on the field and monitored by ADVISES.

The technique presented in this paper is applicable to homogeneous WSNs with static routing topologies. In addition, for illustrative reasons, the specifications presented in the paper focus on node connectivity and isolation problems, and do not take into account the quality of produced sensor data. However, let us stress that the specifications can be easily extended to cover the aspects of WSN behavior not covered in the paper. We will provide specific examples of possible extensions in the paper.

The rest of the paper is organized as follows. A discussion of the related work is given in Section 2. Section 3 is dedicated to the description of a process underlying our the proposed methodology. Section 4 reports the definition of the correctness specifications (general and structural) of WSNs following the *event calculus* formalism. Section 5 addresses the static verification in the form of *robustness checking*. The runtime verification technique is discussed in Section 6. In Section 7 the case studies are discussed. Finally, in Section 8 the paper concludes with final remarks, a discussion of limitations, and indications for future work.

## 2. Related Work

Several approaches have been proposed in the literature for the dependability evaluation of WSNs properties: experimental, simulative, analytical and formal.

Experimental approaches are used to measure the dependability directly from a real distributed system during its operation, and thus they allow the analysis of dependability at runtime [15]. In the field of WSNs, Li and Liu presented in [16] a deployment of 27 Crossbow Mica2 motes that compose a WSN. They describe a Structure-Aware Self-Adaptive WSN system (SASA) designed in order to detect changes of the network due to unexpected collapses and to maintain the WSN integrity. Detection latency, system errors, network bandwidth and packet loss rate were measured; coverage and connection resiliency metrics are not considered. In the prototyping phase, it is possible to perform an accelerated testing, for example by forcing faults in sensor nodes (by means of *Fault Injection* (FI) [17,18]). In [19], a dependability model and a dependable distributed WSN framework for Structural Health Monitoring (called DependSHM) are presented. Another relevant work on dependable WSN for SHM is [20]. An approach is presented in order to repair the WSN and guarantee a specified degree of fault tolerance. The approach searches the repairing points in clusters and places a set of backup sensors at those points. Shakkira et al. [21] propose a lightweight trust decision-making scheme that has also been extended to reduce calculation overheads and improve energy efficiency by integrating an innovative protocol defined for data exchange.

Simulative approaches for assessing WSNs usually make use of behavioral simulators, i.e., tools able to reproduce the expected behavior of a system by means of a code-based description, and they are involved in the design phase. Typical simulative approaches to evaluate WSN fault/failure models are provided in [22,23]. In [22] authors address the problem of modeling and evaluating the reliability of the communication infrastructure of a WSN. The first on-line model-based testing technique [23] has been conceived to identify the sensors that have the highest probability to be faulty. Behavioral simulators, as NS-2 [24] and Avrora [25], allow the reproduction of the expected behavior of WSN nodes on the basis of the real application planned to execute on nodes. However, it is not always possible to observe non-functional properties of WSNs by means of simulative approaches, since models need to be redefined and adapted to the specific network to simulate.

The study of the performance and dependability of WSNs can be performed by means of analytical models [26,27,28,29,30,31]. In [26], authors introduce an approach for the automated generation of WSN dependability models, based on a variant of Petri nets. An analytical model to predict the battery exhaustion and the lifetime of a WSN (LEACH) is discussed in [27]. In [28] the authors present a network state model used to forecast the energy of a sensor. In [32], a methodology to evaluate the reliability and availability of Wireless Sensor Networks in industrial environments that are subject to permanent faults on network devices is proposed. In [30], authors propose a reactive distributed scheme for detecting faulty nodes. In [29], k-connectivity in secure wireless sensor networks under the random pairwise key predistribution scheme with unreliable links is investigated. In [31], a technique to mitigate identity delegation attacks is introduced.

Formal approaches offer new opportunities for the dependability study of WSNs. Recently, different formal methods and tools have been applied for the modeling and analysis of WSNs, such as [33,34,35]. In [33], Kapitanova and Son apply a formal tool to WSNs. They propose a formal language to specify a generic WSN and a tool to simulate it. However, the formal specification has to be rewritten if the WSN under study changes. In [34], Man et al. propose a methodology for modeling, analysis and development of WSNs using a formal language (PAWSN) and a tool environment (TEPAWSN). They consider only power consumption as a dependability metric that is necessary but not sufficient to assess the WSN dependability (e.g., other problems of WSN, such as the isolation problem of a node have been analyzed) and also they apply only simulation. In [35], Boonma and Suzuki describe a model-driven performance engineering framework for WSNs (called Moppet). This framework uses the event calculus formalism [36] to estimate, only at design time, the performance of WSN applications in terms of power consumption and lifetime of each sensor node; other dependability metrics, such as coverage and connection resiliency are not considered. The features related to a particular WSN have to be set in the framework every time that a new experiment starts.

There are some papers ([37,38]) that considered formal methods in real-time contexts. In [37], Olveczky and Mesenguer model and study WSN algorithms using the Real-Time Maude formalism. Though authors adopt this formalism, they use an NS-2 simulator to analyze the considered scenarios, making the work very similar to simulative approaches. The work presented in [38] considers a WSN as a Reactive Multi-Agent System consisting of concurrent reactive agents. In this paper, dependability metrics are not treated and calculated and authors just describe the structure of a Reactive Decisional Agent by means of a formal language.

Finally, in [39], the authors model and evaluate the reliability and lifetime of a WSN under some typical working scenarios based on the sensor node modes (sleep and active) and the mechanism of alternating between these modes. By means of numerical examples, they illustrate the reliability and lifetime of a WSN. Wang et al. [40] implement binary decision diagrams based algorithms to facilitate the design, deployment, and maintenance of reliable WSNs for critical applications.

An open issue with formal specifications of WSNs is that they need to be adapted when changing the target WSN configuration, e.g., in terms of the number of nodes and topology. To address this problem, it is necessary to provide separated specifications and thus conceive two logical sets of specifications: a general specification for WSN correctness properties that is valid for any WSN, and a structural specification related to the topology of the target WSN, designed in order to be generated automatically. Currently, there are proposals in the literature documenting the application of formal methods to model the WSN but they only focus on some dependability metrics as lifetime and power consumption; it is necessary to provide a method of assessing the dependability in terms of other important key dependability metrics, such as coverage and connection resiliency to undesired events. Moreover, no approach has been defined using formal methods for doing static and runtime WSN verification as we propose in this paper.

## 3. The Process of the Methodology

Formal methods have been widely adopted in the literature to verify the correctness of a system, taking into account specifications. The verification is performed by providing proof on an abstract mathematical model of the system. Until now, there is no work that has proven how to use a unique formal approach to perform dependability assessment at design time and at runtime.

This paper defines a new, full, formal methodology to support verification of WSNs both at design time (through *static verification*) and runtime (through *runtime verification*) exploiting only one set of formal specifications divided into two subsets: general (unchangeable and valid for any WSN) and structural (variable on the basis of particular configuration of the WSN) specifications.

**Figure 1:**
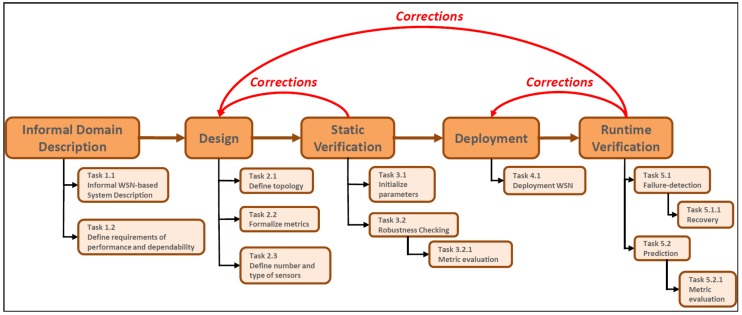
The process of the proposed methodology.

In this Section, we introduce our proposed methodology modeled as a process characterized in steps illustrated in Figure 1.

The process is characterized by five sequential phases: *Informal Domain Description*, *Design*, *Static Verification*, *Deployment* and *Runtime Verification*; the entire process consists of 13 tasks.

### 3.1. Informal Domain Description

In this phase, the application is described and its requirements of performance and dependability (e.g., coverage threshold) are defined.

#### 3.1.1. Task 1.1: Informal WSN-Based System Description

Using natural language, the WSN-based system domain is described in a textual form that is useful to understand what is the considered scenario, what are possible events that can occur, the number of sensors of the network, what they sense, and how many redundant nodes there are.

#### 3.1.2. Task 1.2: Define Requirements of Performance and Dependability

After having defined the application domain, in this task, the performance and dependability characteristics are described. For example, “We want to guarantee at least 65% of coverage for an area against occurred failure events”, “We want the number of failure events active at the same time to be no higher than half of the total number of sensors”.

The dependability metrics considered in this paper, are:

- *connection resiliency* represents the number of node failures and disconnection events that can be sustained while preserving a given number of nodes connected to the sink.
- *coverage* is the time interval in which the WSN can operate, while preserving a given number of nodes connected to the sink.

The computation of these metrics is threshold-based. The threshold expresses the fraction of failed and isolated nodes that the user can tolerate, given its design constraints. For instance, a WSN of 20 nodes and a threshold set to 50% means that, at most, 10 isolated nodes can be tolerated.

The coverage is then defined as the interval [0,t], *t* being the timepoint of the event that caused the isolation of a number of nodes exceeding the threshold.

Clearly, the definition of the threshold depends on the specific problem and relies on expert humans who want to use the approach to make an informed estimation that is useful to improve their design (such as, how to improve the WSN topology in order to make it tolerant to at least 50% of node failures). Nevertheless, while for some applications, a fixed threshold could not be defined, the approach does not restrict the use of a single threshold. Metrics can be evaluated with varying values of thresholds, e.g., to find out the maximum number of tolerable failures to achieve the wanted resiliency level.

It is worth noting that it is easy to extend the set of metrics. Indeed, in some related work, we considered also metrics related with the correct delivery of packets and the power consumption as performance and dependability metrics.

### 3.2. Design

This phase includes the design of a WSN defining the topology represented by means of a tree graph with the sink node as the root. Since several WSNs are composed by sensors that are in a fixed place (i.e., in the case of structural monitoring, hospital environment monitoring, fire monitoring, home environment monitoring, etc), we focus on static routing topologies that can be represented as directed spanning trees [41] (with the sink as the root). Dependability metrics are formalized and number and type of sensors are defined.

#### 3.2.1. Task 2.1: Define Topology

This task defines the topology; nodes and links are identified. From the structure of the WSN, we retrieve the corresponding spanning tree.

#### 3.2.2. Task 2.2: Formalize Metrics

This task formalizes the required metrics on the basis of requirements defined in the previous step (e.g., coverage computation).

#### 3.2.3. Task 2.3: Define Number and Type of Sensors

This task is complementary to the Task 2.1 in order to define topology and fix the number of the sensors. In particular, the properties of sensors -e.g., Receive/Transmit (RX/TX) energy consumption- are defined since they are considered in the next phase (*Static verification*).

### 3.3. Static Verification

The designed WSN is verified in terms of the defined dependability properties. The verification is static in the sense that the network is not still operating.

The effects of the reasoning, performed on the basis of the defined specifications (general and structural), impact on the designed WSN. For instance, if the results of the static verification do not meet the dependability requirements, it could be necessary to modify the topology of the WSN, or relax the requirements.

#### 3.3.1. Task 3.1: Initialize Parameters

This task focuses on the choice and initialization of the parameters needed to perform static verification. The designed topology is loaded; then users can set several parameters, such as number of timepoints, sensors and tolerated failures. Moreover, dependability thresholds are set.

#### 3.3.2. Task 3.2: Robustness Checking

In this task, the WSN design is verified by means of an event-based formal approach. Dependability metrics (formalized in the Section 3.2.2) are evaluated against random sequences of undesired events, useful to identify corner cases and dependability bottlenecks.

#### 3.3.3. Task 3.2.1: Metric Evaluation

This task performs the computation of the dependability metrics (such as coverage and connection resiliency) analyzing the outcome produced by the robustness checking process. The metrics are calculated on the basis of their definitions and considering the output produced by the formal reasoner. Once the metric values have been obtained, this task provides the comparison among them and the threshold values set by the user.

### 3.4. Deployment

In this phase, the WSN, verified at design time, is actually deployed distributing wireless sensors in one or more environments.

#### Task 4.1: Deployment WSN

The aim of this task is to physically deploy the WSN in one or more environments.

### 3.5. Runtime Verification

Runtime verification is performed on the final running system. The aim is to formally check the running system (the WSN) against some correctness properties. The detection of the violation of a correctness property may be used either to trigger a recovery procedure for the running system, or to handle the incorrect status in a safe way. This feature is very useful as long as (although the correctness of the models of the system is granted by formal static verification activities) the quality of the running system may degrade to an unacceptable level, which can be identified just by runtime verification activities.

#### 3.5.1. Task 5.1: Failure-Detection

In this task, failure events (node failures, disconnections, ...), occurring in the wireless sensors, are detected during the system running and an event is generated in a particular formalism in order to start the computing of the new dependability degree.

#### 3.5.2. Task 5.1.1: Recovery

In this task, if the current value of metrics is lower than the desired threshold, the network characteristics (topology, position of the nodes, power of transmission, etc.) can be modified to let the WSN be able to satisfy the required dependability level. For instance, a node X can become a dependability bottleneck if it is positioned in a way that makes it the only one to be used to connect two different portions of the WSN. A failure of node X then makes a whole portion of the WSN isolated. In the recovery task, once the problem is understood, the positions of node X can be modified in order to let it share the traffic load with another node.

#### 3.5.3. Task 5.2: Prediction

This task may allow the designing of predictive models for the running system. In particular, runtime verification is of course mainly focused on identifying the current situation of the system, which is the result of the sequence of events collected until the current moment. However, starting from the current situation and the sequence of past events, it is possible to exploit predictive models to foresee the forthcoming levels of dependability of the system.

#### 3.5.4. Task 5.2.1: Metric Evaluation

This task operates like task 3.2.1. Of course, in this case, the target of the measure is the final running system, instead of a model of it.

## 4. Specifications

In this section, we describe the formal specification of WSN correctness composed by two logical sets: in the first one, we define invariant rules that are applicable to any WSN and thus written only one time; in the second set, we define variable specifications that are dependent on a given WSN structure (i.e., topology, number of nodes, sent packets, etc.). All the defined formal specification underlies the verification process described in the previous section to perform static and runtime verification:

1. *general correctness specification*—set of correctness properties’ specifications, valid independently of the particular WSN under study
2. *structural specification*—a set of specifications and parameters related to the properties of the target WSN, e.g., number of nodes, network topology, quality of the wireless channel (in terms of disconnection probability), and initial charge of batteries. It has to be adapted when changing the target WSN, having thus the advantage of minimizing the effort.

### 4.1. Event Calculus

Since the normal and failing behavior of a WSN can be characterized in terms of an event flow (for instance, a node is turned on, a packet is sent, a packet is lost, a node stops working due to crash or battery exhaustion, or it gets isolated from the rest of the network due to the failure of other nodes, etc.), we adopt an event-based formal language. In particular, among several event-based formal languages, we choose Event Calculus, since its simplicity, its wide adoption in the sensor networks arena [35,42,43,44], and the possibility to formally analyze the behavior of a system as event flows, offer simple ways to evaluate the dependability metrics of our interest, even at runtime.

Event calculus was proposed for the first time in [45] and then it was extended in several ways [46]. This language belongs to the family of logical languages and it is commonly used for representing and reasoning regarding the events and their effects [47].

Fluent, event and predicate are the basic concepts of event calculus [36]. Fluents are formalized as functions and they represent a stable status of the system. For every timepoint, the value of fluents or the events that occur can be specified.

This language is also named “narrative-based”: in the event calculus, there is a single time-line on which events occur and this event sequence represents the narrative. Dependability metrics can be evaluated by analyzing the narrative generated by an event calculus reasoner based on the specification of the target WSN. A narrative is useful to understand a particular behavior of a WSN.

The most important and used predicates of event calculus are:Initiates, Terminates, HoldsAt and Happens.

Supposing that *e* is an event, *f* is a fluent and *t* is a timepoint, we have:

- Initiates (*e*, *f*, *t*): it means that, if the event *e* is executed at time *t*, then the fluent *f* will be true after *t*.
- Terminates (*e*, *f*, *t*): it has a similar meaning, with the only difference being that when the event *e* is executed at time *t*, then the fluent *f* will be false after *t*.
- HoldsAt (*f*, *t*): it is used to tell which fluents hold at a given timepoint.
- Happens (*e*, *t*): it is used when the event *e* occurs at timepoint *t*.

Several techniques are considered to perform automated reasoning in event calculus, such as satisfiability solving, first-order logic automated theorem proving, Answer Set Programming and logic programming in Prolog.

To check the proposed correctness properties defined in event calculus, we use the Discrete Event Calculus (DEC) Reasoner [48]. The DEC Reasoner uses satisfiability (SAT) solvers [49] and by means of this we are able to perform reasoning, such as deduction, abduction, post-diction, and model finding. The DEC Reasoner is documented in detail in [50,51] in which its syntax is explained (e.g., the meaning of the symbols used in the formulas).

### 4.2. General Correctness Specification

The general correctness specification is described in the following. It specifies that a WSN performs correctly if no undesired events (or failures) happen. From the results of a detailed Failure Modes and Effect Analysis (FMEA) conducted on WSNs in [52], the following are examples of undesired events, ordered from the most severe one to the least severe one:

1. isolation event, i.e., a node is no longer able to reach the sink;
2. packet loss event, i.e., a packet is lost during the traversal of the network;
3. battery exhaustion event, i.e., a node stops working since it has run out of battery.

These first two types of events are actually not independent, but might be caused by simpler “basic events”, such as the stop of one or more nodes (e.g., due to crash or battery exhaustion), or the temporary disconnection of a node to its neighbor(s) due to transmission errors. So, the occurrence of the third event (battery exhaustion) might cause packet losses and isolation events, if no alternative routes are present in the network. In turn, the battery exhaustion event is dependent on the power consumption of the nodes as a consequence of packet sending and receiving activities (in general assumed to be power demanding activities with respect to CPU activities [53]).

In this paper, we concentrate on the specifications related to the first event (isolation event) being the most severe one according to the FMEA in [52]; specifications of the other two events (packet loss and battery exhaustion), defined also in [52], are here omitted for a matter of space.

**Figure 2:**
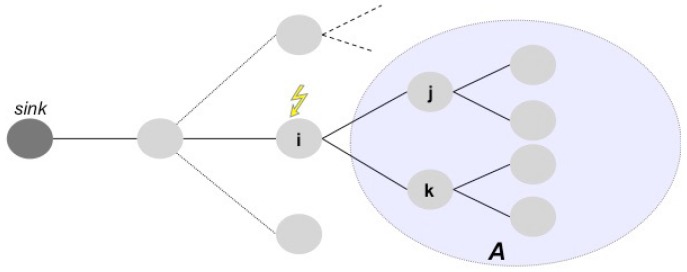
Isolation of a Wireless Sensor Network subnet.

The isolation event happens when a node is no longer able to reach the sink of the WSN, i.e., the gateway node where data are stored or processed. For instance, considering the WSN represented in Figure 2, if node *i* fails, then nodes *j*, *k*, and in general all the nodes belonging to the subnet *A* become isolated.

More in general, if a subnet depends on a node and this node stops or becomes isolated, then all of the nodes of the subnet are isolated.

In Table 1, we report the basic elements (sorts, events and fluents) used for the specification of situations like these. We distinguish basic events from generated events. These last events are generated by the reasoner on the basis of the specification and of the sequence of basic events which actually occurred.

Listing 1 shows the rules that represent the core of the specification for an isolation event. In lines 1–7, we define a rule to verify when a node becomes isolated. A sensor can be isolated if it is initially reachable, alive and, considering a link with another sensor, there is no other sensor that is alive, reachable and connected with the isolated sensor.

Also, we report (in lines 9–14) another rule which allows the checking of a Join event. In particular, a sensor that is isolated can rejoin the network and become reachable again if it still is alive, its neighbor sensor is alive and reachable from the sink node and a connection between them has been restored.

In lines 16–28, we show conditions in which Isolation and Join events cannot occur. We claim that an Isolation event cannot occur in a sensor (in lines 16–21) if at least one of its neighbor sensors is alive and reachable and is connected with the sensor. Moreover, if a sensor is not reachable, due to a previous isolation, or not alive, it cannot receive another Isolation event again. In the similar way (in lines 23–28), if a sensor is reachable or not alive or all of its neighbor sensors are not alive or not reachable, then it cannot join to the network, remaining isolated.

Listing 1 **Correctness Specification for the Isolation event**


[1]  [sensor, from_sensor, time] Neighbor (from_sensor, sensor) & HoldsAt (
[2]      IsReachable (sensor), time) & HoldsAt (IsAlive (sensor), time) &
[3]      (! {from_sensor2} (HoldsAt (IsAlive (from_sensor2), time) &
[4]      HoldsAt (IsReachable (from_sensor2), time) & HoldsAt (
[5]      IsLinked (sensor, from_sensor2), time)) &
[6]      Neighbor (from_sensor2, sensor)) −>
[7]  Happens (Isolate (sensor), time).
[8]
[9]  [sensor, from_sensor, time] ( !HoldsAt (IsReachable (sensor), time) &
[10]     HoldsAt (IsAlive (sensor), time) & HoldsAt (IsAlive (
[11]     from_sensor), time) & HoldsAt (IsReachable (from_sensor), time))
[12]      & HoldsAt (IsLinked (sensor, from_sensor), time) &
[13]     Neighbor (from_sensor, sensor) −>
[14]  Happens (Join (sensor), time).
[15]
[16]  [sensor, from_sensor, time] ((HoldsAt (IsAlive (from_sensor), time) &
[17]     HoldsAt (IsReachable (from_sensor), time) & HoldsAt (
[18]     IsLinked (sensor, from_sensor), time)) | !HoldsAt (
[19]     IsReachable (sensor), time) | !HoldsAt (IsAlive (sensor), time))
[20]      & Neighbor (from_sensor, sensor) −>
[21] !Happens (Isolate (sensor), time).
[22]
[23] [sensor, from_sensor, time] ( HoldsAt (IsReachable (sensor), time) |
[24]     !HoldsAt (IsAlive (sensor), time) | !HoldsAt (
[25]     IsLinked (sensor, from_sensor), time) |
[26]     !HoldsAt (IsAlive (from_sensor), time) | !HoldsAt (
[27]     IsReachable (from_sensor), time)) & Neighbor (from_sensor, sensor)−>
[28] !Happens (Join (sensor), time).

### 4.3. Structural Specification

General correctness specifications are complemented by a structural specification that comprises a set of specifications and parameters related to the properties of the target WSN, e.g., number of nodes, network topology, quality of the wireless channel (in terms of disconnection probability), and initial charge of batteries. This specification depends on a particular WSN topology and thus, differently from the specifications described in the previous sub-section, it varies on the basis of the characteristics of the target WSN.

**Figure 3:**
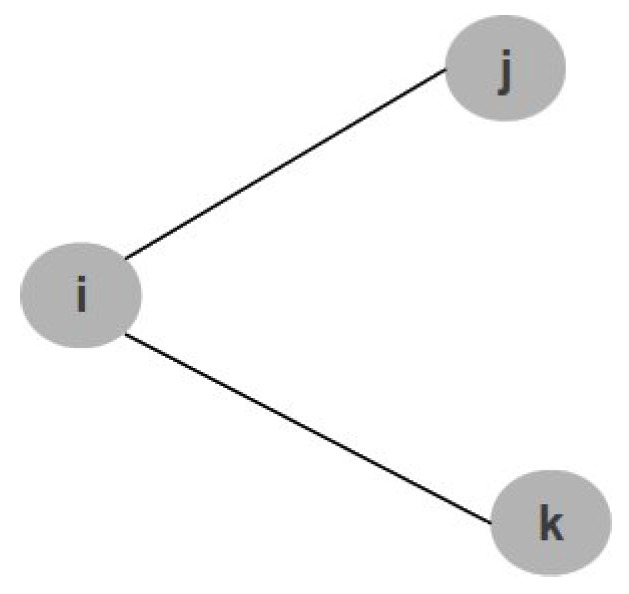
Example of topology of a WSN.

To specify the topology, we use the predicate Neighbor (already used in the previous specifications) to indicate how nodes are linked in the topology. For instance, considering the topology in Figure 3: node *i* is connected with *j* and *k* and the sink (root node) is the node *i*.

The resulting specification is reported in Listing 2, where *sensor 1* is the parent node (*i*) and *sensor 2* is child nodes (*j* and *k*). Clearly, this specification can be changed easily if the topology of the WSN changes.

Listing 2 **Use of the Neighbor predicate in a structural specification**


[1]  [sensor1, sensor2] Neighbor (sensor1, sensor2)  <−>  (
[2]  (sensor1 = i & sensor2 = j)  |
[3]  (sensor1 = i & sensor2 = k)
[4]  ).

The role of the Neighbor predicate is very important to understand when an axiom can be applied. Let us examine the axiom related at a possible isolation (lines 1–7 of listing reported in Listing 1) and let us apply it for Figure 3. The described implication is true when, given a couple of nodes (sensor, from_sensor), the conditions about isolation are true and there is a link between nodes (in this case, between node *j* and *i* or between node *k* and *i*). This, for instance, can never be true for the couple of nodes *j* and *k*, since there is not a physical link between them.

Regarding the parameters, their values can be used to check the correctness properties of the WSN under different conditions, i.e., under different assumptions on the initial charge of batteries (e.g., to verify a WSN in the middle of its life), or under different environmental conditions affecting the quality of the channels (impacting on the probability of having a disconnection event when checking the robustness of the WSN).

### 4.4. Metrics Computation

The metrics of interest can be evaluated by using the narrative produced by the reasoner starting from the specification.

Starting from the coverage, it can be calculated by considering the threshold value and by analyzing the IsReachable(sensor) and IsAlive(sensor) fluents found to be true in the event trace produced by the reasoner: if a IsReachable(x) or a IsAlive(sensor) fluent is false in the event trace, this means that node *x* became isolated or it stopped. For example, in the case of coverage at 50%, for a WSN with seven nodes, there is coverage when at least four nodes are not isolated (i.e., they are reachable). Hence, as soon as four different nodes are neither reachable nor alive (looking at the fluents), the network is not covered anymore. The coverage can be then evaluated as the interval [0,t], *t* being the timepoint of the last failure or disconnection event in the narrative before the isolation (e.g., the timepoint of the event that caused the isolation of a number of nodes exceeding the threshold).

The connection resiliency can then be evaluated as the number of failure and disconnection events (namely, Stop(sensor) and Disconnect(sensor, from_sensor) events) that happen within the coverage interval, excluding the last failure/disconnection event, that is, the one that actually leads the number of isolated nodes to overcome the threshold. For example, if we have coverage in the interval [0, 6], and during this period three failure/disconnection events can be counted, then the connection resiliency is 2, that is, the WSN was able to tolerate two failures or disconnections while preserving more than 50% of the nodes connected.

Let us stress that the specifications and metrics adopted in the paper are chosen for illustrative reasons to show the use of the proposed methodology for static and runtime verification in practical terms. If needed in particular application settings, the specification can be easily extended with more fluents and then the narrative used to evaluate other metrics. For instance, one can add a ”battery level” fluent to evaluate the power consumption of nodes, or a ”packet delivery” fluent to model the flow of packets among nodes and evaluate the probability of correct packet delivery.

### 4.5. Example: A Wireless Body Sensor Network

**Figure 4:**
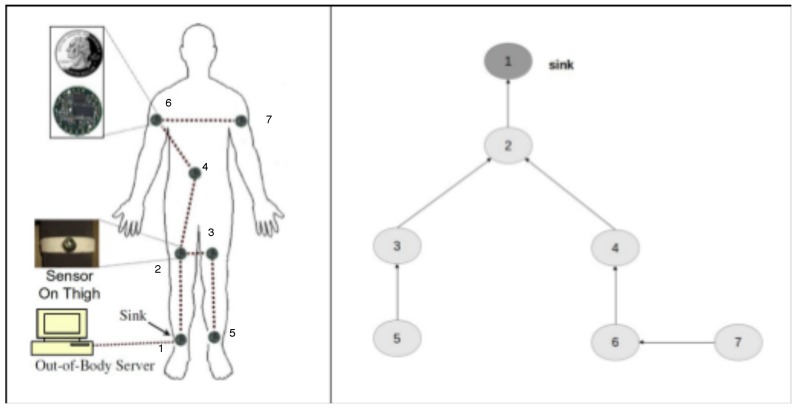
Quwaider wireless body sensor network (WBSN) and related topology.

Let us consider a simple example to show the use of the specification on a WSN and how the narrative produced by the event calculus reasoner is useful to compute the metrics of interest. In particular, we consider a wireless body sensor network (WBSN) realized by Quwaider et al. [54] and illustrated in Figure 4.

This WBSN (Figure 4 on left side) is constructed by mounting seven sensor nodes attached on two ankles, two thighs, two upper-arms and one on the waist area. Each node consists of a 900 MHz Mica2Dot MOTE (running Tiny-OS operating system).

On the right side of Figure 4, we report the node tree graph corresponding to the WSN, where the arrows indicate the relationship between a couple of nodes (i.e., node 2 depends on node 1, node 3 and 4 depend on node 2, etc.).

The corresponding specification of the topology is reported in the Listing 3.

Assuming a coverage threshold of 50%, let us suppose to be interested in analyzing the behavior of the WBSN if the following events occur: Disconnect(5,3) at timepoint 1 and Stop(4) at timepoint 3.

Listing 3 **Structural specification of the WBSN topology**


[6] [sensor1, sensor2] Neighbor (sensor1, sensor2) <−> (
[7] (sensor1 = 1 & sensor2 = 2) |
[8] (sensor1 = 2 & sensor2 = 3) | (sensor1 = 2 & sensor2 = 4) |
[9] (sensor1 = 3 & sensor2 = 5) |
[10] (sensor1 = 4 & sensor2 = 6) |
[11] (sensor1 = 6 & sensor2 = 7)
[12]).

If the specification is correct, we should observe a coverage interval that equals to [0,3] (i.e., when node 4 stops at timepoint 3, four nodes are not reachable, namely 4, 5, 6 and 7), and a connection resiliency equals to 1 (i.e., only one event—the Disconnect (5,3) event—is tolerated). To test the desired event sequence, we add an event trace (Listing 4) to the specification, composed by a list of Happens predicates that specify nodes and timepoints in which a given event occurs. The completion statement specifies that a predicate symbol (i.e., Happens) should be subject to predicate completion.

Listing 4 **Event trace**


[14]  Happens (Disconnect (5, 3), 1).
[15]  Happens (Stop (4), 3).
[16]
[17]  completion Happens
[18]

Finally, in the last part, we consider ranges of values for sensors and timepoints (Listing 5). In this case, we know that the network is composed of seven nodes and we want to observe what could happen in 10 timepoints.

Listing 5 **Parameters**


[21] range sensor 1 7
[22] range time 0 10

Listing 6 reports the outcome (the narrative) produced by the DEC Reasoner. The event trace confirms our expectations. We can observe that after the stop of node 4, nodes 6 and 7 become not reachable. Considering that node 5 was already not reachable, this means that a total of four nodes are isolated. The coverage is computed as the timepoint of the last failure event causing such isolation, that is 3. Consequently, the connection resiliency is computed by counting the number of failure and disconnection events in the interval [0,3], excluding the last event; hence, it is equal to 1, as expected.

For this example, we consider both the coverage and connection resiliency threshold set as values chosen without particular meaning; in fact, on the basis of the desired outcome, the thresholds can be set by means of the our proposed tool.

Listing 6 **Outcome of the DEC Reasoner**


[1] 0
[2] 1
[3] Happens (Disconnect (5, 3), 1).
[4] 2
[5] -IsLinked (5, 3).
[6] Happens (Isolate (5), 2).
[7] 3
[8] -IsReachable (5).
[9] Happens (Stop (4), 3).
[10] 4
[11] -IsAlive (4).
[12] Happens (Isolate (6), 4).
[13] 5
[14] -IsReachable (6).
[15] Happens (Isolate (7), 5).
[16] 6
[17] -IsReachable (7).
[20] 7
[21] 8
[22] 9
[23] 10

## 5. Static Verification

In the previous example, we have shown how the specifications and the reasoning performed on them can be exploited to analyze the WSN response to a given sequence of undesired events. This concept can be extended to test the WSN against a variable sequence of events, in order to verify its design (textitstatic verification) in the form of a coverage robustness checking.

Specifically, the verification consists of analyzing the robustness of the network, in terms of coverage, against a variable number of failures (stop and disconnection events), from 1 to *n*, where *n* is selected by the user, considering all combinations without repetitions. This is useful to check how many node failures the network can tolerate, while guaranteeing a given minimum level of coverage. For example, if we consider a network composed by *m* nodes and a threshold coverage equal to 50%, we may want to understand what are the sequences of failures causing more than *m*/two nodes to be isolated (i.e., coverage under the specified threshold) and how the resiliency level varies when varying the sequences of failures. This allows the evaluation of the maximum (and minimum) resiliency level reachable by a given topology and what are the critical failure sequences, i.e., the shortest ones causing a loss of coverage. These are particularly useful to pinpoint weak points in the network (so-called dependability bottlenecks).

We developed an algorithm to generate automatically the sequences of failures (stop and disconnection events specified with Happens predicates) against which to check the robustness of the WSN. The algorithm is implemented by the ADVISES tool (see Section 7.1), and it is aimed at reducing the number of failure sequences to be checked. The principles are to avoid repetitions and to end the sequence as soon as the coverage level becomes lower than the user defined threshold. For instance, we start considering all the cases when there is one failure. By means of the DEC Reasoner, we compute the coverage; if the coverage is above the threshold, the resiliency is surely greater than 1, because there is just one failure and it is tolerated in all cases. In the generic *k*-th step, we consider sequences of *k* failures. If the generic sequence $\left\{f_{1},f_{2},...,f_{k}\right\}$ leads to a coverage below the threshold, we do not consider sequences starting with an $\left\{f_{1},f_{2},...,f_{k}\right\}$ prefix in the (k+1)-th step. By considering the percentage of sequences with *k* failures where the coverage is above the threshold, let us say $r_{k}\%$, we can say that the resiliency is *k* in $r_{k}\%$ of cases.

## 6. Runtime Verification

The aim of this step is i) to perform a Runtime Verification (RV) [12,55] detecting failure events occurring in a real WSN, possibly to activate recovery actions and ii) to perform a prediction of the critical levels of next failure events that may occur in the WSN, in order to take countermeasures in advance. In this case, critical events, e.g., Stop and Disconnect, are not simulated anymore with Happens predicates, but they are detected from the real system, through system monitors. Details about such monitors are not in the scope of this paper, and are addressed in our previous work in [56].

An application scenario is considered to show how the runtime verification can be implemented; the aim is to describe how it is possible to catch events and observe their effects in a WSN at runtime.

**Figure 5:**
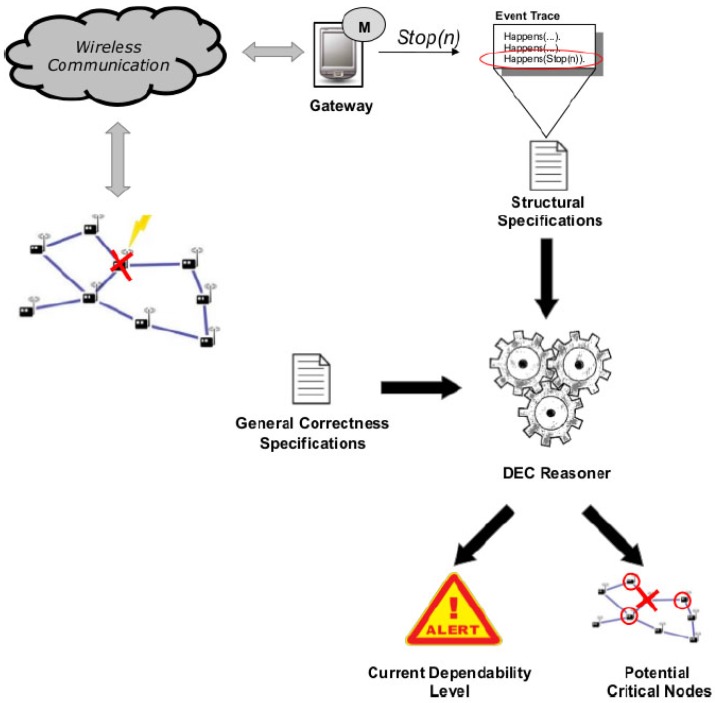
Application scenario in runtime context.

We can see, in Figure 5 from the left to right, that when an event occurs in a wireless sensor node, it is detected by a system monitor that runs on a gateway. We assume that the gateway is a more powerful and stable device with respect to single WSN nodes. While this assumption is usually satisfied in practice (WSN gateways are usually dedicated computers, directly powered and stably connected to the Internet), the gateway itself can become a single point of failure for the method. This problem, not addressed in this paper, can however be mitigated by deploying more than one gateway.

The failure event of a WSN node (for instance Stop(n)) is managed by the monitor deployed on the gateway and added to the current event trace to perform the reasoning. The new event trace is included in an updated structural specification; thus, the DEC Reasoner receives the structural specification with the last occurred event and, considering the general correctness specifications (initially defined), performs the reasoning, returning a couple of outcomes: (i) the Current Dependability Level of the WSN and (ii) the Potential Critical Nodes.

The first outcome reports the current WSN dependability level (i.e., the WSN now covers the *X*% of the monitored area, and it has been resilient to *Y* failures so far). The second outcome is a prediction about possible critical events that may occur after the current event (e.g., from now on, node *Z* represents a weak point in the WSN: it should be replicated or its batteries should be replaced). Moreover, the runtime verification is useful to further verify, at runtime, the WSN design that has been validated at design time. Even if a WSN is checked at design time, it is necessary to observe whether the implemented WSN conforms to expectations and to continuously monitor whether it is able to cope with unexpected events. If the network becomes isolated due to the failure of the only node connecting to the gateway, then the whole network will result isolated. This severe failure event could be already recognized by the method at design time, either by performing a what-if analysis or a robustness checking run, suggesting that the user reinforce the connectivity of the network.

Clearly, the quality of the information provided to the user depends on the quality of the detection. However, let us stress that the consequences of a wrong detection can be mitigated by the user. The outcome of the runtime verification is an alert to the user coupled with an indication of potential critical nodes. The alerted user can then check the actual state of the network before performing any inadequate reaction. Let us also observe that the probability of a wrong detection depends on the type of events to be detected. In the case of a fail stop behavior (such as a node crash as assumed in the paper) then the probability of wrong detection is very low since it is easy to verify for a given interval of time (through the monitors) if the node is indeed stopped. In this case, it is possible to provide reliable information to the reasoner and to the user.

## 7. Case Studies

In this section, by means of two case studies, we apply the methodology described in Section 3 to study and improve the robustness of two WSNs.

We have performed our experiments on a Intel P4 machine, CPU Clock 3.5 GHz, 512 MB RAM, equipped with Linux, Kernel 3.8.8. Although rather old, this hardware setting is representative of the processing power of a hypothetical gateway to be used at runtime (see Section 7.2.2).

We have focused on WSN-based healthcare systems due to their criticality related to patient monitoring scenarios.

In Table 2, we report an analysis of studied papers focused on WSN-based healthcare systems in order to select the most interesting topologies for our case studies on the basis of the number of nodes and the kind of topology (tree, grid, fully-connected, ...).

After general research, we have chosen the following systems: the Self-powered WSN for remote patient monitoring (adopted in hospitals) [61] and the *MEDiSN* [59] (a WSN for automating process of patient monitoring in hospitals and disaster scenes).

The two networks have features that are useful to check the performances of our tool and thus collect interesting results. The former (Self-powered WSN) is represented by a perfect balanced tree; the latter is characterized by a sequence of three nodes in a row that could easily cause the majority of bottleneck and isolation problems; in particular, considering the MEDiSN network, we show how this network could improve by modifying the placement of the nodes.

In order to facilitate and automatize the application of the proposed methodology (comprising static and runtime verification) against a general case study, a Java-based tool, called ADVISES (available on sourgeforce) (AutomateD VerIfication of wSn with Event calculuS),has been designed and implemented.

### 7.1. The ADVISES Tool

The goal of the ADVISES tool [52] is to provide technical support to the methodology addressing practical aspects (e.g., setting of parameters necessary to start verification, realization of structural specification in an automatic way and merge with general specification).

This tool has been realized (i) to operate in double mode: static and runtime; (ii) to automatically generate the structural specifications given the properties of a target WSN; (iii) to perform the reasoning starting from the correctness and structural specifications; (iv) to compute dependability metrics starting from the event trace produced by the reasoner; and (v) to receive events in real-time from a WSN to start runtime verification and to evaluate current and future criticalities.

In particular, at runtime, it is like a server that is in waiting for new events coming from the WSN and that are detected by means of a system monitor.

Since the static and runtime verification do not require the same number of input parameters, we present the ADVISES tool operating in double mode: in static mode we need to select several parameters that in runtime mode they are not necessary.

#### 7.1.1. ADVISES for Static Verification

The ADVISES tool in static verification needs several parameters, such as the number of packets, the number of failures, the battery capacity value of a node, the initial event trace, etc.

**Figure 6:**
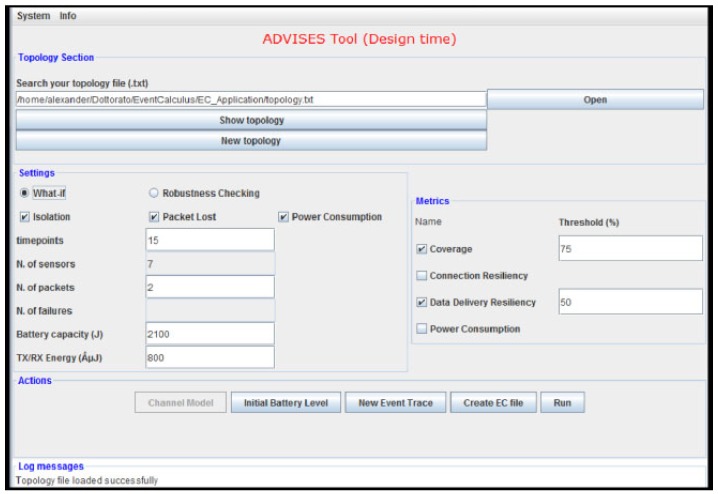
Outcome of ADVISES in static mode running. Parameters for What-if analysis and Robustness Checking computing are available.

By means of the interface shown in Figure 6, a user can simply specify (i) the topology of the target WSN (using a connectivity matrix); (ii) the formal correctness specifications (e.g., for checking isolation events); (iii) the temporal window size to consider (in terms of the number of timepoints); (iv) the number of packets that each sensor can send; (v) the number of failures to be simulated (in case of robustness checking); (vi) the battery capacity of a sensor (in J) and the needed energy for RX/TX operations (in *μ*J); (vii) the metrics to calculate (for coverage, the threshold value is also necessary); (viii) the channel model; (ix) the initial battery level (to simulate nodes that do not start with full battery capacity; if not specified, then the battery capacity (parameter vi) is assumed as initial battery level); (x) the initial event trace (in case the user wants to perform a “what-if” analysis for a given set of events instead of a robustness check with random sequences of undesired events).

The number of failures to be simulated is a parameter that can be set by the user depending on his/her objectives. For instance, if the user wants to verify whether the WSN is resilient to at least three failures (connection resiliency equals 3) then at least three failures have to be simulated for each robustness checking random run. Clearly, the number of failures should not exceed the number of nodes or the number of paths present in the topology. However, this check is currently not performed by the tool.

The interface is subdivided by four panels:

- “Topology” section is dedicated at the loading/creating of a network topology. The user has to select a topology file that can be found by means of an “Open” button or created on-the-fly using the “New topology” button. The “Show topology” button allows the display of the selected topology in a separate frame. In this file, links between nodes are represented;
- By means of “Settings”; the user can set the several parameters (number of timepoint, of packets, of failures, battery capacity and RX/TX energy) that will be considered for the static verification; the number of sensors is automatically calculated by the ADVISES tool from the topology file. Moreover, the user can choose what general correctness specifications (e.g., the “Isolation” shown earlier) are necessary for a particular verification. Other specifications, such as Packet Lost or Power Consumption, are already available in the tool, but are not treated in the present paper.
- In the “Metrics” section, the user with a tick chooses the desired metrics and if he selects coverage or data delivery resiliency, he has to write the percentage values (default value is 50);
- In the fourth panel (*Actions*), the ADVISES tool presents the possible steps that the user can perform;
  - “Channel Model”
    Pressing this button, the users can select the disconnection probability for every link of the WSN topology choosing a percentage value (0% = never disconnection; 100% = always disconnection) in order to simulate temporary losses of connectivity between nodes. This action is disabled if the user wants to perform a what-if analysis (disconnections in this case will be directly inserted in the event trace);
  - “Initial Battery Level”
    Pressing this button, the users can set the initial battery level for every node of the WSN topology, choosing a percentage value (0% = completely discharged node; 100% = completely charged node).
  - “New Event Trace”
    Pressing this button, the users can set the initial event trace that could occur in the WSN, in the case of a what-if analysis (disabled in the case of robustness checking).
  - “Create EC File”
    Pressing this button, the ADVISES tool automatically generates an Event Calculus file in the same directory as the topology file. This file wraps the general correctness specifications chosen in the “Settings” section and the structural specifications. Once the EC file is created, it is displayed in a separate frame;
  - “Run”
    To obtain the output of the DEC Reasoner, the user has to press this button; analyzing the fluent values contained in the outcome produced by the DEC Reasoner, the ADVISES tool computes the desired dependability metrics. A pop-up message will appear on the screen in order to notify the user of the end of the computation;
  - “Log Messages”
    Panel, the ADVISES tool reports all the useful messages in order to inform the user if some error occurs.

From the list of parameters, it can be noted that some of them are very fine grained (e.g., energy for transmissions), but on the other hand, some coarse grained parameters, which could also have a significant impact on the performance and dependability, are missing (e.g., size of transmitted messages). This depends on the fact that the Event Calculus specification adopted in the paper is used as a proof-of-concept. Let us however point out that the tool is extensible with more specifications, in case we want to include more aspects in the verification (e.g., size of messages or different metrics such as power consumption). In this case, the tool also has to be modified in order to take an extended set of parameters as input.

#### 7.1.2. ADVISES for Runtime Verification

The ADVISES tool in runtime verification mode is simpler because it works on the basis of the events that it receives from the WSN; there are less computations to perform. In fact, at design time, having chosen a number of timepoints to observe, the tool has to calculate many combinations of events; the more timepoints we consider, the more combinations of events are obtained.

The timepoints (in the use of Event Calculus) are a set of natural numbers (0, 1, 2, ...) which are used to define a sequence of events. There is no relation between timepoints and the real time, and the timepoints may also assume a different temporal nature (seconds, minutes, hours, ...) into the same study.

Instead, at runtime, the ADVISES tool for each timepoint is waiting for an event to compute the effects of this occurred event.

**Figure 7:**
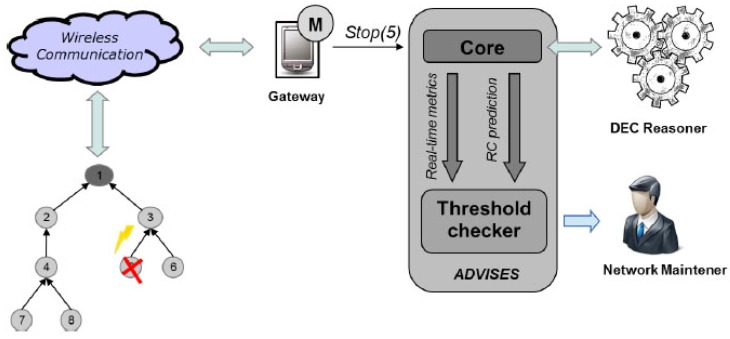
Outcome of ADVISES in runtime mode running. Only timepoint parameter is editable: this is the observation time in an experiment at runtime.

Figure 7 shows the ADVISES operating at runtime.

Once started, it is in server mode and waiting for events coming from the WSN; when a failure occurs in a wireless sensor node, (for instance Stop(5)), it is managed by the monitor and sent to the ADVISES Tool that is listening on a port ready to receive events and start reasoning. Having received the event, it automatically updates the sequence of received events and generates the event calculus specification file in order to perform the reasoning with the DEC Reasoner. The ADVISES tool, having received the output from the DEC Reasoner, calculates the current values of the selected dependability metrics to establish the current status of the WSN and performs robustness checking to predict the future criticalities. Then, having verified the obtained metrics values with the thresholds set by the user, the ADVISES tool sends messages to a network maintainer which may be purely informative, or alerts in case these values are under the desired threshold. After the last step, the ADVISES tool continues to work, waiting for the next detected events.

**Figure 8:**
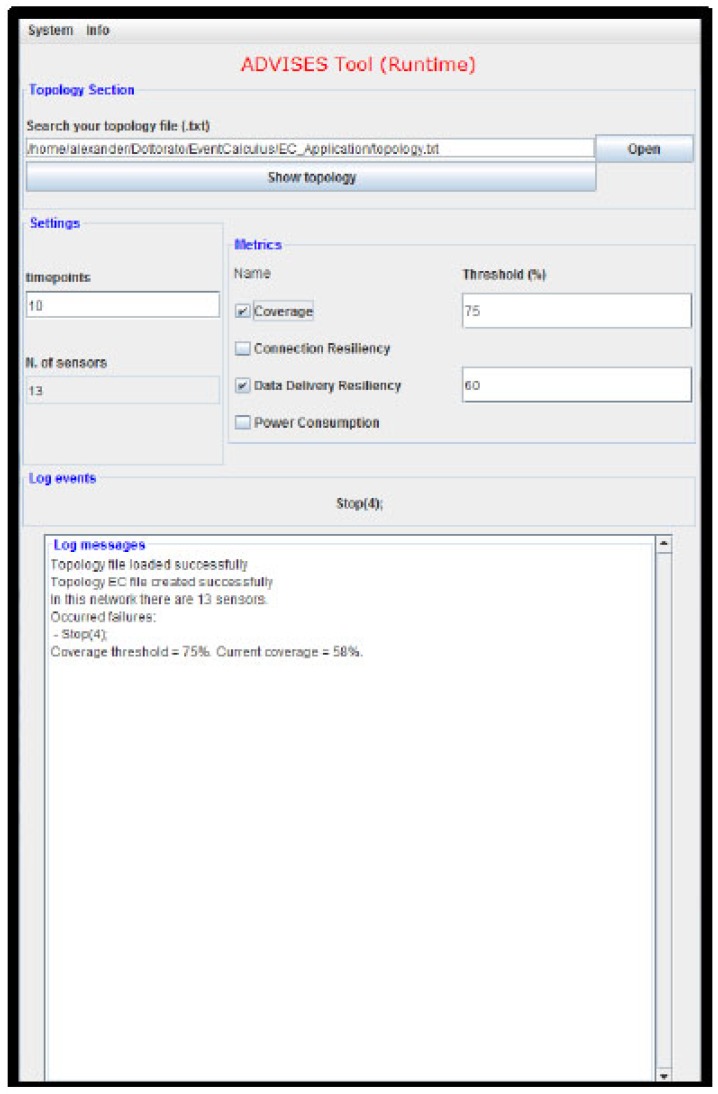
ADVISES in runtime mode.

Figure 8 shows this interface.

By means of this interface, a user can simply specify (i) the initial topology of the target WSN, using a connectivity matrix; (ii) the temporal window size to consider, in terms of the number of timepoints to consider for the prediction; (iii) the metrics to calculate. Actually, we have to set an initial topology since a wireless sensor network could be affected by some changes due to the nature of the sensors: a wireless sensor could stop and change its neighbors. This concept is also illustrated in the Case Studies section in which we propose improvements of the initial topologies considered. The tool itself does not directly manage topology changes at runtime, but it provides indications to the user upon failures on how to manage the WSN and improve the topology in order to be more resilient in case of further undesired events. From this point, the user can run the tool with the new topology chosen after the failure.

In the “Log messages” panel, the ADVISES tool reports all the performed computations about the current state of the WSN and the risks that may affect its robustness. Also, there are useful messages in order to inform the user if some error occurs.

**Figure 9:**
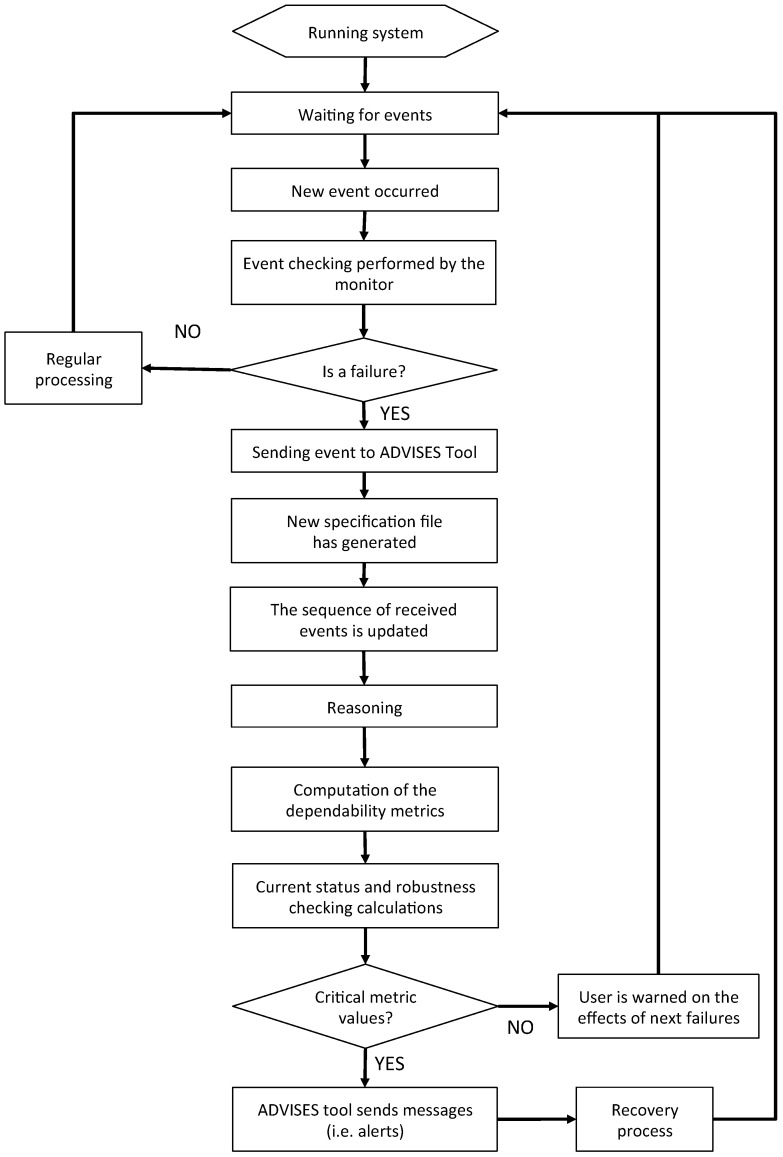
Runtime verification process illustrated by a flow diagram.

In Figure 9, we describe the runtime verification process (performed by the ADVISES tool) by means of a flow diagram.

Therefore, the ADVISES tool advises the network maintainer of problems related to the network and reports its critical points.

For this purpose, we have realized another interface of the ADVISES tool in order to receive events from a WSN in real-time detected through a system monitor and to start the runtime verification, evaluating both current and future criticalities.

### 7.2. Experimental Results

We perform two sets of experiments. For the first one, we consider the self-powered WSN [61] that is composed by 13 motes: one base station (that consists of a mote and is connected to a server), four router nodes (realized utilizing CrossBow MICAz motes) and eight sensor nodes (such as ECGs, pulse-oximeters, etc.). For the second one, we consider MEDiSN [59] that is composed of 10 motes: one base station (as gateway), four relay points (that are wireless sensors) and finally five physiological monitors (collecting patients’ physiological data). For this network, we have considered a topology in which physiological monitors in the leaves are treated as if they were relay points as well, so that the topology is more complex and interesting to try our system with.

For each topology, we try to improve the networks balancing the nodes: in this way, there are more possibilities of having no isolation events and more coverage in case of some failure nodes. For our study, the ADVISES Tool helps to quickly compute the metrics of dependability by means of us being able to apply improvements.

#### 7.2.1. Static Verification

For both networks, we aim to observe the percentage of cases in which there is connection resiliency to 1, 2 and 3 failures, keeping a coverage threshold at 65%, 75%, and 85%, so from a less demanding to a more demanding resiliency requirement. Moreover, starting from the original topologies of the two networks as presented in [59,61], we attempt to make them more robust, reconfiguring the connections among the nodes and observing effects.

**Figure 10:**
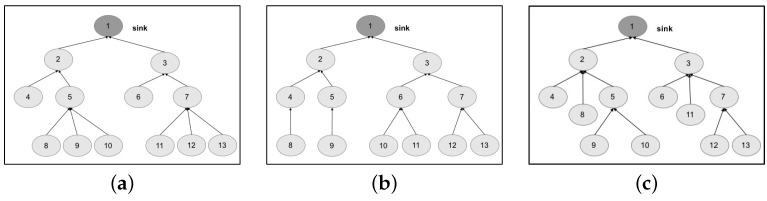
Topologies of the self-powered WSN. (**a**) Original; (**b**) First attempt; (**c**) Second attempt.

**Figure 11:**
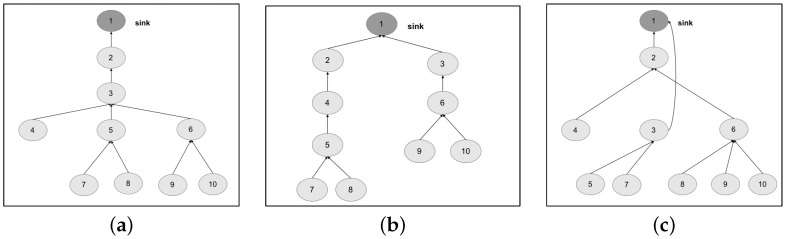
Topologies of MEDiSN. (**a**) Original; (**b**) First attempt; (**c**) Second attempt.

We design the topologies, related to the two chosen networks, by means of a tree graph. The first topology is structured as a tree graph with three levels (Figure 10a); the second topology (Figure 11a) is structured as a tree graph with four levels.

Exploiting the capabilities of the ADVISES tool, we specify the characteristics of both networks and of the metrics to be evaluated; specifically, connection resiliency with different coverage thresholds. In Table 3, we present results of the robustness checking: on the columns, we identify the topologies (original and our proposed alternatives) grouped by the network (self-powered WSN and MEDiSN); on the rows, we collect the results of connection resiliency for one, two and three failures on the basis of coverage threshold (65%, 75% and 85%); the generic cell of the table represents the robustness of the network, evaluated as the percentage of cases in which the topology (identified by the column) is able to tolerate *n* failures (where *n* corresponds to the value of the connection resiliency identified by the row) guaranteeing a certain coverage value (identified by the row).

Results on column Topology 1 are achieved by performing robustness checking and dependability evaluation of original topologies. We can observe that, if we increase the requirement on connection resiliency (from 1 to 3) and on coverage (from 65% to 85%), the percentage of cases in which the WSN is able to tolerate the failures decreases, as expected. We can also note that the self-powered WSN is more robust than the MEDiSN one, due to the higher number of nodes and the lower number of levels of the tree graph.

Starting from these results, we try to improve the robustness of both networks, by means of slight changes on the network topology. The aim is to show how the proposed approach and related tool are useful to drive design choices and tune the configuration of the network. Considering the self-powered WSN, we start by proposing a more uniform distribution of the nodes, balancing the tree. The resulting topology is shown in Figure 10b. What we expect to observe is a general increase of robustness, since a failure of a node causes the disconnection of a smaller number of nodes with respect to the original topology (e.g., see nodes 5 and 7). For a coverage threshold at 65%, we achieve this conclusion (see column Topology 2 in Table 3). However, opposite to what was expected, for a coverage threshold at 75% (and three failures) and a coverage threshold at 85% (and two failures), we obtain a degradation of robustness, from 27% to 18% and from 43% to 27%, respectively.

The automatic analysis performed by the reasoner on the specifications allow us to deeply investigate the exact reason of such degradation, and find better solutions. To this aim, in Table 4, we report a classification of the failure events tested by the ADVISES tool on the self-powered WSN. The events are grouped based on the obtained coverage value (reported in the Coverage column) for each of the topologies considered, and are also grouped as below or above the 75% coverage threshold. Passing from Topology 1 to Topology 2, we can observe that Topology 2 is able to reduce the overall number of events causing a coverage value below the threshold (critical events), if compared to Topology 1, improving the resiliency in several cases. However, we also note that Topology 2 introduces a new criticality, not present in Topology 1, related to node 3: if this node fails or gets disconnected, the coverage suddenly drops down to 47%. Hence, node 3 represents a dependability bottleneck for Topology 2. Guided by this result, we propose another slight modification to the topology, obtaining a new version (Topology 3) shown in Figure 10c, reducing both the number of child nodes of node 3 and the path length of nodes 8 and 11. In this case, looking at the Topology 3 column in Table 4, we can observe that the criticality on node 3 disappears, while reducing the overall number of critical events compared to Topology 1. The benefits of this new topology are also visible in Table 3; specifically, topology 3 is able to improve the robustness in all cases with respect to Topologies 1 and 2.

In a similar way, we exploit our approach to find better topologies for the MEDiSN case study. In this case, we have observed that if we keep the same number of tree levels but reduce the number of leaf nodes (see the topology in Figure 11b), the robustness level decreases, as shown in the Topology 2 column in Table 3. Looking at Table 5 (reporting the classification of critical events achieved for the MEDiSN WSN), we can note that, even if Topology 2 is able to reduce the criticality of nodes 2 and 3, which are clearly two dependability bottlenecks for the original MEDiSN topology, it introduces new critical events (e.g., the failure of node 4). Moved by these results, we increase the number of leaf nodes and decrease the tree levels, obtaining a third topology (Topology 3 in Figure 11c) able to reduce the number of critical events (see Topology 3 column in Table 5) and to improve the overall resiliency level in all cases (see the Topology 3 column in Table 3).

In both the cases, we have seen how the proposed approach is useful to precisely spot dependability bottlenecks and define more robust configurations. For both experiments, the ADVISES tool has performed a total of 161,460 reasonings on the specifications (114,480 for the self-powered WSN and 46,980 for MEDiSN). In particular, Table 6 shows the number of failure sequences and the related time spent by the ADVISES Tool to perform the reasoning in the self-powered WSN and in MEDiSN networks considering a coverage threshold value equal to 75%. In the worst case (12,144 different failure sequences tested for the self-powered WSN) the tool takes about 9 h to perform the evaluation on our commodity hardware. While this time could still be acceptable at design time (and it does not affect the conceptual validity of the approach), it clearly represents a practical limit, especially for large WSNs. This issue, and related solutions that we are currently investigating, are further discussed in Section 8.

#### 7.2.2. Runtime Verification

After the static analysis, we physically deploy the best topologies (topology 3 for both WSNs) at our labs to perform runtime verification.

For this purpose, we have designed and implemented a system monitor with the aim of detecting failure events from the real-world WSN. The monitor runs on a machine and listens for packets coming from all sensors trough the sink node of the WSN. The detection of events (such as the stop of a node) is performed assuming that each sensor sends packets periodically, with a known rate, which is common to several WSN applications. Hence, for every node, the monitor sets a timeout, which is reset each time the monitor receives a packet from the given node. If the timeout expires for a node X, the monitor sends a Stop (X) event to ADVISES (running in runtime mode). The use of time-out may also detect temporary disconnections or delays. In this case, when packets from a node X are received again after a stop, the monitor sends a Start (X) event to ADVISES. Clearly, different failure detection approaches could be used as well, however this is not relevant for our experiment and out of the scope of the paper.

The monitor has been implemented as a java application running on a server (a Pentium 4 machine in our case) and connected via USB to a MIB520 Base station by Crossbow. As sensor nodes, we have adopted Iris Motes by Crossbow equipped with a ZigBee RF Transceiver and a TinyOS 2.0 operating system, running the BlinkToRadio application, just to perform a periodic sensing and sending of packets of all nodes to the sink.

Considering the deployment of third self-powered WSN topology, we start the system monitor that is in communication with the ADVISES Tool.

**Figure 12:**
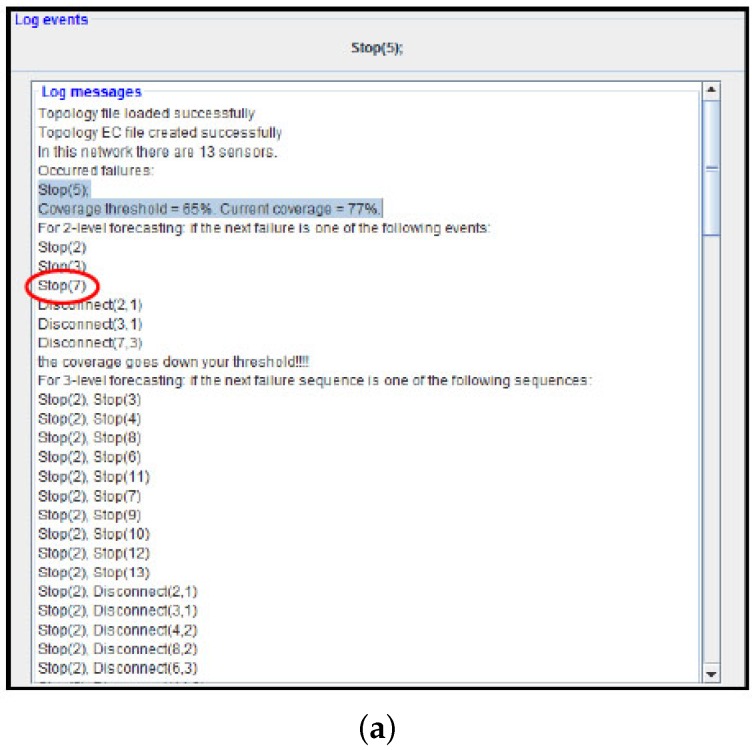

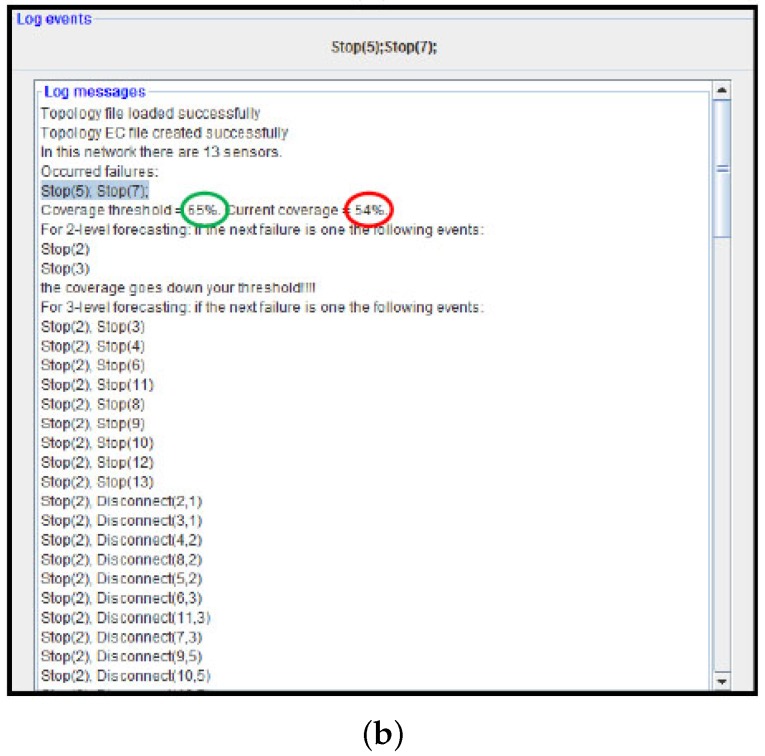
Outcomes of an example of the ADVISES Tool when it is set in Runtime Verification mode. (**a**) Current coverage value is 77% (against the coverage threshold value 65%) in the case of node 5 failure and warnings for next Stop events; (**b**) Current coverage value is 54% (against the coverage threshold value 65%) in the case of both node 5 and 7 failures; in this case coverage is under the threshold value set.

As a first test, we stop node 5. The failure event is detected by the system monitor at runtime (elapsed time ≈ 1 s) and it is sent to ADVISES. Once the failure event has been received, ADVISES starts the reasoning and shows the result in the “Log message” panel as described in Figure 12a (metric computation time ≈ 1 min and 30 s): coverage is equal to 77%. ADVISES also identifies potential critical nodes (prediction phase) that could compromise the robustness of the network; in fact, from Figure 12a, we can see that the tool “advises” the user (prediction time ≈ 4 m) that if the next failure event that occurs is, for example, Stop (7), then the network is not robust anymore (the coverage goes below the desired threshold, 65% in this test). To validate this suggestion, we also stop sensor node 7. From the log posted by the ADVISES tool (Figure 12b), we observe that effectively the coverage decreases (54%) and goes under the desired threshold.

From the experiment, we can note that the adoption of the approach to monitor a real WSN at runtime is straightforward. Using the same formal specifications used at design time, the tool is able to tell, at runtime, what is the current dependability level of the WSN (e.g., coverage at 77%) and to pinpoint critical nodes (e.g., nodes 2, 3 and 7 in the example) that need to be maintained (e.g., by replacing batteries) or strengthened (e.g., by replicating them with other nodes) to avoid the whole mission of the WSN being compromised.

## 8. Conclusions and Future Work

This paper addressed the problem of the dependability assessment of WSNs with formal methods. Assessing the dependability of WSNs is a crucial task since these kinds of networks are more and more used in critical application scenarios where the level of trust becomes an important factor; depending on application scenarios, different dependability requirements can be defined, such as, node lifetime, network resiliency, and coverage of the monitoring area. From a preliminary analysis, the need emerged for verification of a WSN at design time in order to increase the confidence about the robustness of designed solutions before putting it into operation; and the need for monitoring a WSN during operation in order to avoid unexpected results or dangerous effects and thus to perform what in the literature is defined as continuous monitoring.

The research activity dealt with the definition of formal specifications used for the behavioral checking of WSN-based systems at design and runtime phases; a set of correctness specifications applied to a generic WSN has been defined using event calculus as formal language since the behavior of a WSN can be characterized in terms of an event flow (e.g., a node turns on, a packet is sent, a node stops due to failure, etc.) and the event calculus formalism allowed the easy specification of the system in terms of events.

This paper demonstrated through proof-of-concept scenarios that it is possible to assess the dependability of WSNs by means of formal methods, in particular event calculus formalism, using the narrative it generates. By means of two case studies, we have shown how the adoption of a formal specification is helpful to deeply investigate the reasons for inefficiencies, in terms of the degree of dissatisfaction of given dependability requirements, and to suggest viable improvements to the design that have positive effects on the global dependability level of the system. The implementation of a tool, namely ADVISES, has also shown how the approach can be easily adopted by technicians with no experience of formal methods, the structural specification being generated automatically and completely hidden to users. Finally, the paper described how the same specification can be adopted to perform continuous monitoring at runtime, once the designed WSN is implemented and deployed on the field.

The implementation and actual use of the approach also helped us to highlight practical limits to be solved. We have considered topologies of WSNs adopted in typical critical scenarios, such as healthcare (with 7/15 nodes). The simplicity of such topologies, although taken from real examples, may lead to doubts regarding the general usefulness of the approach (the same reasoning could be performed intuitively with no need of a tool). However, we selected the examples with the aim of pointing out how ADVISES allows the quantitative justification of design choices against precise requirements and metrics, helping technicians to have the instruments to make informed design decisions, e.g., when he/she is forced to choose between a few topologies to be considered, due to restrictions. The selection has been done also with the objective of implementing the topology on the field, with real WSN nodes, in order to practically show how the same approach and tool can be used both at design time and at runtime for continuous monitoring.

Concerning the time complexity of the method, it is strictly dependent on that of the EC, which has been estimated (assuming *n* the number of events) as O($n^{-3}$) in case of absolute time and total ordering of the events [65], O($2^{n-3}$) in the case of relative times and partial ordering [66]. The proposed methodology relies on two kinds of analysis (runtime verification and static verification) based on relative times and partial ordering of events. Therefore, the time complexity is upper bounded to O($2^{n-3}$). For runtime verification, the reasoner is invoked just one time at the occurrence of the adverse event in the network. For static verification, the reasoner is invoked a number of times, corresponding to the combinations without repetitions of the emulated failures. Generally, in both cases, the number of events k generated for the analysis in EC is the square of the number of nodes N of the network.

During the experimental phase, we have also tested the correct functioning of the specification with topologies with more nodes (about 100) but we experienced a remarkable deterioration of the performance on our commodity hardware (about 1 h to test all the sequences with only one failure), due to the state space explosion problem. While this issue does not affect the conceptual validity of the approach and its use on typical critical WSNs, it undermines its practical adoption for very large WSNs. The issue can be partially solved at design time by using more powerful hardware and by considering that the user, at design time, is more favourable to waiting for the reasoning result if this can help his/her design decisions. However, the issue remains for the runtime monitoring, where the output of the reasoning must be provided with due timing constraints and with no assumptions on the processing power of gateways. To face this scalability problem, we are currently conceiving a method to divide a large topology in several sub-topologies (equivalent to WSN clusters). During the runtime, the different sub-topologies can be monitored by different gateways (acting as cluster heads), performing the reasoning on each sub-topology in parallel and then finally joining the results taking into account the dependences between the sub-topologies.

Although the methodology here presented may not always identify an optimal topology from all possibly desirable features, it still improves the support to analyze similarly complex topologies where the difference may not be obvious to the designer. The generic strategy and tool presented in this article facilitates the experimentation and comparison of different heuristics to address different problems. Two obvious areas of experimentation and improvement are the relaxation of restrictions and the improvement of efficiency. From this perspective, the computational platform used for experimentation in this article is anecdotal and the main contribution is in problem solving strategies.

We base our approach on sensors that are fixed (such as beacons) and with an established data routing reducing a topology, such as a spanning tree that is valid for a WSN. As future work, we plan to extend the use of the specification also for mobile scenarios, specifying further events that notify wireless sensor movements within clusters and from cluster to cluster.

## Figures and Tables

**Table 1:** Basic elements of the specification for the isolation event.

Elements	Name	Description
**Sorts**	sensor	Reference sensor for events and fluents
	to_sensor	Sensor used in case of connection (i.e., a sensor connects to another sensor)
	from_sensor	Sensor used in case of disconnection (i.e., a sensor disconnects from another sensor)
**Basic Events**	Start (sensor)	Occurring event when a sensor turns on
	Stop (sensor)	Occurring event when a sensor turns off
	Connect (sensor, to_sensor)	Occurring event when a sensor connects to another sensor
	Disconnect (sensor, from_sensor)	Occurring event when a sensor disconnects from another sensor
**Generated Events**	Isolate (sensor)	Occurring event when a sensor is isolated from the network
**Fluents**	IsAlive (sensor)	True when a Start event occurs for a sensor
	IsLinked (sensor, to_sensor)	True when a Connect event occurs
	IsReachable (sensor)	True when a sensor is reachable from the sink node
	Neighbor (sensor1, sensor2)	True when sensor 1 is directly linked to sensor 2

**Table 2:** Analysis of some WSN healthcare systems.

Work	Nodes	Topology	Sensor Platform
iNODE-based system [57]	4	tree	iNODE
BSN-based system [58]	8	fully-connected	Jennic JN5139
MEDiSN [59]	10	tree	Sentilla Tmote Mini
HM4ALL [60]	12	tree	JN5139-MOI ZigBee-based platform
Self-powered WSN [61]	13	tree	Crossbow Micaz
Multi-patient system [62]	15	grid	Tmote sky
CodeBlue [63]	16	grid	N.A.
Clinical Monitoring System [64]	18	tree	TelosB mote

**Table 3:** Percentages of connection (Conn. Resil.) resiliency for the self-powered WSN and MEDiSN. Topology 1 is the original network, Topology 2 and 3 are attempts of coverage (see “Outcome”) improvement. Three thresholds of coverage (Cov. = 65%, 75% and 85%) have been selected.

Outcome		Self-Powered WSN			MEDiSN		
		Topology 1	Topology 2	Topology 3	Topology 1	Topology 2	Topology 3
Cov. = 65%	Conn. Resil. = 1	83%	83%	83%	77%	66%	77%
	Conn. Resil. = 2	52%	61%	67%	41%	30%	49%
	Conn. Resil. = 3	31%	35%	41%	18%	10%	28%
Cov. = 75%	Conn. Resil. = 1	66%	83%	83%	55%	44%	66%
	Conn. Resil. = 2	43%	48%	50%	29%	18%	43%
	Conn. Resil. = 3	27%	18%	29%	0%	0%	0%
Cov. = 85%	Conn. Resil. = 1	66%	66%	66%	55%	44%	66%
	Conn. Resil. = 2	43%	27%	43%	0%	0%	0%
	Conn. Resil. = 3	0%	0%	0%	0%	0%	0%
Examined failure sequences		114,480			46,980		

**Table 4:** Classification of failure events for a self-powered WSN (Topology 1, 2 and 3).

	Failure Event			Coverage
	Topology 1	Topology 2	Topology 3	
Coverage < 75% (critical events)	no event failure	Stop(3) OR Disconnect(3,1)	no event failure	47%
	Stop(2) OR Stop(3) OR Disconnect(2,1) OR Disconnect(3,1)	no event failure	Stop(2) OR Stop(3) OR Disconnect(2,1) OR Disconnect(3,1)	54%
	no event failure	Stop(2) OR Disconnect(2,1)	no event failure	62%
	Stop(5) OR Stop(7) OR Disconnect(5,2) OR Disconnect(7,3)	no event failure	no event failure	70%
Coverage ≥ 75%	no failure event	Stop(6) OR Stop(7) OR Disconnect(6,3) OR Disconnect(7,3)	Stop(5) OR Stop(7) OR Disconnect(5,2) OR Disconnect(7,3)	77%
	no failure event	Stop(4) OR Stop(5) OR Disconnect(4,2) OR Disconnect(5,2)	no failure event	85%
	Stop(4) OR Stop(6) OR Stop(8) OR Stop(9) OR Stop(10) OR Stop(11) OR Stop(12) OR Stop(13) OR Disconnect(4,2) OR Disconnect(6,3) OR Disconnect(8,5) OR Disconnect(9,5) OR Disconnect(10,5) OR Disconnect(11,7) OR Disconnect(12,7) OR Disconnect(13,7)	Stop(8) OR Stop(9) OR Stop(10) OR Stop(11) OR Stop(12) OR Stop(13) OR Disconnect(8,5) OR Disconnect(9,5) OR Disconnect(10,5) OR Disconnect(11,7) OR Disconnect(12,7) OR Disconnect(13,7)	Stop(4) OR Stop(6) OR Stop(8) OR Stop(9) OR Stop(10) OR Stop(11) OR Stop(12) OR Stop(13) OR Disconnect(4,2) OR Disconnect(6,3) OR Disconnect(8,5) OR Disconnect(9,5) OR Disconnect(10,5) OR Disconnect(11,7) OR Disconnect(12,7) OR Disconnect(13,7)	93%

**Table 5:** Classification of failure events for MEDiSN (Topology 1, 2 and 3).

	Failure Event			Coverage
	Topology 1	Topology 2	Topology 3	
Coverage < 75% (critical events)	Stop(2) OR Disconnect(2,1)	no event failure	no event failure	10%
	Stop(3) OR Disconnect(3,2)	no event failure	no event failure	20%
	no event failure	no event failure	Stop(2) OR Disconnect(2,1)	40%
	no event failure	Stop(2) OR Disconnect(2,1)	no event failure	50%
	no event failure	Stop(3) OR Stop(4) OR Disconnect(3,1) OR Disconnect(4,2)	Stop(6) OR Disconnect(6,2)	60%
	Stop(5) OR Stop(6) OR Disconnect(5,3) OR Disconnect(6,3)	Stop(5) OR Stop(6) OR Disconnect(5,4) OR Disconnect(6,3)	Stop(3) OR Disconnect(3,1)	70%
Coverage ≥ 75%	Stop(4) OR Stop(7) OR Stop(8) OR Stop(9) OR Stop(10) OR Disconnect(4,3) OR Disconnect(7,5) OR Disconnect(8,5) OR Disconnect(9,6) OR Disconnect(10,6)	Stop(7) OR Stop(8) OR Stop(9) OR Stop(10) OR Disconnect(7,5) OR Disconnect(8,5) OR Disconnect(9,6) OR Disconnect(10,6)	Stop(4) OR Stop(5) OR Stop(7) OR Stop(8) OR Stop(9) OR Stop(10) OR Disconnect(4,2) OR Disconnect(5,3) OR Disconnect(7,3) OR Disconnect(8,6) OR Disconnect(9,6) OR Disconnect(10,6)	90%

**Table 6:** Reasoning time of the ADVISES Tool for a self-powered WSN and MEDiSN considering a threshold value equal to 75%.

Connection Resiliency	Self-Powered WSN		MEDiSN	
	Failure Sequences	Elapsed Time (s)	Failure Sequences	Elapsed Time (s)
1	24	1500	18	600
2	552	9720	306	2160
3	12,144	32,580	4896	2820
